# Unleashing the pandemic volatility: A glimpse into the stock market performance of developed economies during COVID-19

**DOI:** 10.1016/j.heliyon.2024.e25202

**Published:** 2024-01-28

**Authors:** Umar Nawaz Kayani, Ahmet Faruk Aysan, Mrestyal Khan, Maaz Khan, Roohi Mumtaz, Muhammad Irfan

**Affiliations:** a College of Business, Al Ain University, Abu Dhabi P.O. Box 122612, United Arab Emirates; b Hamad Bin Khalifa University, Qatar Foundation, Non-Resident Fellow Middle East Council on Global Affairs (ME Council), Research Associate, the University College London, Centre for Blockchain Technologies (UCL CBT), Qatar; c Department of Management Sciences, Balochistan University of Information Technology, Engineering, & Management Sciences (BUITEMS), Quetta, Pakistan; d COMSATS University Islamabad, Islamabad, Pakistan; e Department of Leadership Management & Human Resources (LMHR), International Business School, Teesside University, Middlesbrough, TS1 3BX, UK

**Keywords:** COVID-19, Volatility, Developed markets, Stock indices, GJR-GARCH model

## Abstract

The COVID-19 pandemic has resulted in significant financial losses globally, increasing the volatility of financial assets. Thus, this study models the stock market volatility of developed economies during the COVID-19 pandemic. For this purpose, we used the GJR-GARCH (Albulescu, 2020; Albulescu, 2020) [1,1] econometric model on the daily time series returns data ranging from 01^st^-July-2019 to 18th-November-2020. The entire dataset was equally divided into two subsets; before COVID-19, and after the COVID-19. The empirical results of this study showed the presence of volatility clustering, leverage effect, and excess kurtosis indicating leptokurtic phenomena in all stock indices returns. In addition to this, it can be noted that compared to before COVID-19, the stock markets showed negative returns, and increased volatility during the COVID-19. Hence, based on these findings, this study provides significant insights for global stock market investors and economic policymakers regarding financial portfolio construction particularly during crises times.

## 1 Introduction

The Coronavirus (COVID-19) outbreak, which started to spread in Wuhan (city of China) initially, soon transformed into a global pandemic. As of March 11, 2020, the disease had infected over 1 million people worldwide and resulted in thousands of deaths [1]. As a result, World Health Organization (WHO) declared it a global pandemic. The number of cases and deaths continued to rise, with 169,691,118 infected and 3,526,708 deaths as of May 28, 2021 (WHO, 2021). The pandemic has also caused widespread fear and panic, creating a sense of emergency across the globe [2].

Furthermore, COVID-19 has caused a bearish trend in the global stock markets. Moreover, the stock market acts as a barometer for the economy and is a key indicator of the capital wealth of a nation. During this deadly virus, the stock markets have shown an increase in fear factor among investors to invest in stock markets hence, causing heavy financial losses across the globe. Therefore, it is essential to analyze the stock market volatility during the period of crisis. Particularly, when the basics of financial instruments such as stocks are diluted by macroeconomic factors is a big threat [3]. In the beginning, the disease spread only in China, but with each passing day, the disease spread across Italy, France, Germany, the United States, Australia, etc.

In comparison with other financial crises and pandemics, COVID-19 has caused economies confronted with several challenges. Due to the fact that we live in an integrated world in terms of economies [4]. The ongoing pandemic can destroy any economy if not managed properly [5,6]. The countries adopting severe lockdown strategies amid COVID-19 have resulted in a substantial economic recession, and heavier economic prices and now becoming a matter of life [7]. Likewise, it affects the transportation industry as well [8], and commodities [9].

Moreover, financial markets globally experienced significant investment losses because of the COVID-19 pandemic [[10], [11], [12], [13], [14], [15], [16], [17]]. The smooth flow of funds is mainly done via stock exchanges across the world. Stock market prices are influenced by the economic variables [18]. Subsequently, the performance of stock markets can have an immediate influence on the economy. Stock market predictions usually address the risks associated with the stock market investment.

Researchers relate the volatility of the stock returns with the uncertainty in the stock market. Therefore, volatility is the key parameter in making investment decisions in different asset classes. Furthermore, volatility is the utmost risk indicator thus accurate forecasting of stock market volatility is significant [19]. Greater volatility refers to the higher fluctuations in the return's series in the short run which means an increase in the volatility is an increase in the risk level. Whereas, lower volatility shows that stocks have low fluctuations in their returns or price series in the shorter run, and prices change at a stable rate over time [20]. Additionally, the higher volatility relates to the increase in the probability of a bearish trend in the market whereas, lower volatility increases the chances of a bullish trend in the market [21].

In this paper, we have modeled the stock market volatility of developed economies across the globe during the COVID-19 crisis. Furthermore, the study contributes to the existing body of knowledge by investigating the volatility behavior of 21 developed stock markets. Secondly, the study is novel in terms of modeling the volatilities of the selected stock market in both before, and during the COVID-19 period. Thirdly, the study has examined the reactions of developed markets to the COVID-19 pandemic along with the assessment of the market trends.

Furthermore, the motivation for conducting this research stems from the recognition of the substantial financial losses and increased volatility witnessed worldwide during the COVID-19 pandemic. Given the unprecedented nature of this crisis, there is a pressing need to gain a comprehensive understanding of the patterns and characteristics of stock market return volatility in developed economies. By employing the GJR-GARCH [1,1] model, this study seeks to provide valuable insights into the behavior of stock market returns before and during the pandemic, with a specific focus on volatility, leverage effects, and leptokurtic phenomena. The research findings are expected to offer significant implications for global stock market investors, empowering them to make well-informed decisions regarding portfolio construction strategies in times of crisis. By examining the unique impacts of the COVID-19 crisis on developed stock markets, this study aims to advance the existing body of knowledge and contribute to the development of effective risk mitigation and opportunity identification approaches during periods of substantial market disruption. Based on these arguments we hypothesize that the volatility of developed stock markets increased during the COVID-19 crisis.

Moreover, this study focuses on investigating the relevance of the COVID-19 pandemic in shaping the volatility of stock market returns in developed economies. Employing the GJR-GARCH [1,1] model, the research examines the extent of volatility exhibited by these markets during the pandemic period. The findings indicate a notable impact, characterized by negative returns, increased volatility persistence, leptokurtic phenomena, and a leverage effect within developed stock markets. These outcomes emphasize the significance of the COVID-19 crisis in shaping financial markets and offer valuable insights for global investors seeking to construct portfolios during times of crisis. By analyzing stock market volatility, identifying negative returns, investigating volatility persistence and leptokurtic phenomena, and exploring the leverage effect, this study provides comprehensive understandings of the behavior of developed stock markets during the COVID-19 pandemic, contributing to the knowledge of effective crisis-time portfolio construction strategies.

The remaining portion of the paper is divided into the following sections: Section 2 represents literature review, section 3 comprises the data and methodology, section 4 refers to the empirical findings. Whereas the conclusion is given in section 5.

## 2 Literature review

Recent financial literature documents that during the widespread of COVID-19 pandemic, the stock returns declined whereas the stock market volatility increased significantly [[22], [23], [24], [25]]. Also, the empirical evidences suggest that the government actions in response to the COVID-19 pandemic could alleviate the volatility of the equity markets [26,27]. Moreover, the implementation of the lockdowns policy resulted in deteriorating the stock market liquidity and stability [28,29].

COVID-19 has significantly affected the stock markets of the several countries across the globe. Likewise, studies proved that sharp decline in the returns of the stock markets and increase in their volatilities, globally [[30], [31], [32], [33], [34], [35]]. Similarly, Khatatbeh, Hani [30] investigated the reaction of eleven equity markets of different countries infected by COVID-19. They found that the equity returns of those stock markets drastically declined when the news of COVID-19 was spread among the general public. Also, Alzyadat and Asfoura [36] examined the impact of COVID-19 event on the Saudi Arabian stock market. Their results showed exponential decline the in the returns of Saudi Arabian stock market during the spread of COVID-19.

Apart from affecting the stock markets, COVID-19 pandemic has also influenced other markets such as commodities, and cryptocurrencies. For instance, Kumar, Kumar [37] in their study investigated the causality among prices of gold, crude oil, bitcoin, and stocks during the COVID-19 pandemic. They found the presence of long run cointegration among the selected assets during the COVID-19 except bitcoin. Likewise, Kumar, Singh [38] using EGARCH model investigated the volatility spillovers across the prices of natural gas, crude oil, exchange rate, gold, and stock market. Their results revealed that the energy commodities do not affect the Indian stock market whereas, exchange rate significantly affected the stock market volatility. Similarly, Kumar, Kumar [39] investigated the causal relationship among crude oil, gold, exchange rate, and stock market. Based on NARDL method, they found that crude oil prices have a significant positive effect on the Indian stock market, and exchange rate negatively influences the stock market in the short run. Also, the gold prices had no impact on the stock market.

Volatility refers to the barometer of measuring the total risk that investors is confronted when investing in the financial assets. Furthermore, volatility is considered as the significant factor in making decision related to investment portfolio management [40]. Whereas, stock market volatility is the systematic risk taken by the investors due to holding market portfolios [41]. Mostly, stock market volatility dramatically increases during the significant events which can have enormous economic and financial effects, for example Asia economic crisis (1997–1998), and recently COVID-19 crisis. Moreover, abnormal increase in the stock market volatility increases negative investor sentiments regarding the future economic developments [42]. According to Schwert [43] stock market volatility represents the uncertainty of future cash flows. In another study, Mazur, Dang [44] empirically revealed that non-symmetrical volatility is negatively related to stock returns.

Moreover, standard deviation, skewness, and kurtosis are the most common measure of volatility. The drawback of using standard deviation is that it assumes of normal distribution of returns however, actual returns are asymmetrically distributed. In contrast, skewness focuses on the extreme data points rather than concentrating on the average returns [45]. Besides, kurtosis is another measure that incorporates extreme values in a dataset [46].

Mostly, financial time series data exhibit three types of behaviors namely; volatility clustering, fat tail phenomena, and leverage effect, these characteristics differentiate financial time series returns analysis from that of other classes of assets data. Hence, during the period of the financial crisis, the stock market returns volatility cannot accurately be modeled through traditional measures such as standard deviation and variance. Therefore, time-varying or dynamic volatility models are required [47]. However, GARCH family models possess the characteristics of incorporating the time-varying volatility. Thus, due to their accuracy, GARCH families are consistently used in the financial literature for the purpose of modeling volatility in the financial time series data [48]. Thus, after extensive review of the financial literature this study is novel in terms of modeling the stock market volatility in times of COVID-19 pandemic for developed economies in one single study.

## 3 Data & methodology

### 3.1 Data

For the purpose of investigating the volatility of developed stock markets, the study has incorporated the stock indices returns data of 21 developed stock markets ranging over the period of 01-July-2019 to 18-November-2020. The study has downloaded the data from www.investing.com. The entire data set was divided into two categories. 01-July-2019 to 10-March-2020 was considered as “before the COVID-19 period”, whereas 11-March-2020 to 18-November-2020 was regarded as the “during the COVID-19 period.” The period for the two phases was selected based on the day of the announcement of COVID-19 as a global pandemic which is 11-March-2020 declared by the World Health Organization (W.H.O). Moreover, the list of developed stock markets along with their symbols is provided in Table 1.

**Table 1:** List of developed stock markets and their ticker codes.

S.No.	Stock Market	Ticker Code	S.No.	Stock Market	Ticker Code
1	Australia	ASX 200	12	Netherlands	AEX
2	Austria	ATX	13	New Zealand	NZX 50
3	Belgium	BEL 20	14	Norway	OSE Benchmark
4	Canada	S&P/TSX	15	Portugal	PSI 20
5	Denmark	OMXC20	16	Spain	IBEX 35
6	Finland	OMX Helsinki 25	17	Sweden	OMXS30
7	Germany	DAX	18	Switzerland	SMI
8	Hong Kong	FTSE China 50	19	United Kingdom	FTSE 100
9	Ireland	ISEQ All Share	20	United States	Dow 30
10	Israel	TA 35	21	France	CAC 40
11	Japan	Nikkei 225			

Furthermore, to investigate the general characteristics of the dataset, the study has calculated the descriptive statistics (mean, standard deviation, skewness, and kurtosis). Besides, Jarque-Bera test was applied to figure out the distribution of stock market returns. In addition to this, all the empirical analysis was performed on the stock returns computed from the formula of continuous compounding, as illustrated in equation (1).

(1) $$R_{t}=\ln\left(\frac{P_{t}}{P_{t-1}}\right)$$

The symbol $R_{t}$ refers to the stock returns at time period t, log natural function is represented by ln whereas, $P_{t}$ shows the price of a stock at the current time period, and $P_{t-1}$ denotes the stock price at previous time period.

### 3.2 Unit root test

Basically, the unit root test is used to confirm whether the data series is stationary or not. Time-series data is said to possess the characteristics of stationary i.e., if the shift in the time does not affect the pattern of the time series data distribution, and if the series is stationary, it shows that mean, variance, and autocorrelation structure remain static over the period of time. To check the stationarity in the time series, Augmented Dickey-Fuller test was applied, which includes a null hypothesis as the presence of unit root [49]. Furthermore, equation (2) below illustrates the ADF test.

(2) $$\Delta y_{t}=\propto_{0}+\theta y_{t-1}+\sum_{i=1}^{n}\propto \Delta y_{t}+e_{t}$$

Where y refers to the time series, t shows the time period, n denotes the optimum number of lags, $\propto_{0}$ is the constant term, and e is called the error term.

### 3.3 ARCH effect test

ARCH-LM abbreviated as Auto-Regressive Conditional Heteroscedasticity-Lagrange Multiplier test was applied to confirm the presence of heteroscedasticity and ARCH/GARCH effect for residuals [50]. Furthermore, equation (3) mathematically represents the ARCH-LM expression.

(3) $$u_{t}^{2}=\gamma_{0}+\gamma_{1}u_{t-1}^{2}+\gamma_{2}u_{t-2}^{2}+\ldots +\gamma_{p}u_{t-p}^{2}+v_{t}$$

Where u is the residual square, which can be computed by the basic regression model. However, p lags are included in a secondary regression model.

### 3.4 GJR-GARCH model

Glosten, Jagannathan [51], proposed the extension of GARCH known as the “GJR-GARCH model”, which incorporates the leverage effect in the financial time series data. Alongside, GJR-GARCH models the volatility clustering and fat tail phenomena. The leverage effect assumes that investors react more toward the negative shocks as compared to the positive ones even if their magnitude is the same. Thus, GJR-GARCH [1,1] model comprises the additional term for the leverage effect represented by gamma (γ). The leverage term equals one when the conditional variance is negative, and it becomes zero when the conditional variance is negative.

Moreover, both GARCH, and GJR-GARCH approaches are often used in the financial and time series literature. However, the results extracted from GJR-GARCH model outperforms the simple vanilla GARCH specifications due to the fact that GJR-GARCH specifications include leverage effect [52,53]. To analyze the research primarily aims to analyze and model the volatility of stock market returns during the COVID-19 pandemic in developed economies. As such, the focus is more on understanding the magnitude and characteristics of volatility rather than explicitly exploring the interdependencies and correlations between different assets. Therefore, opting for a simpler and more focused model, such as the GJR-GARCH [1,1] model, allows for a more direct examination of volatility patterns during the specific crisis period of interest [20]. Hence, this study employed the GJR-GARCH model in order to investigate the stock market's volatility during the COVID-19 pandemic. Furthermore, the mathematical representation of the GJR-GARCH [1,1] model is given in equation (4).

(4) $$\sigma_{t}^{2}=\omega +\alpha_{1}u_{t-1}^{2}+\beta_{i}\sigma_{t-1}^{2}+\gamma_{i}I_{t-1}\;u_{t-1}^{2}$$

In the equation above $I_{t-1\;}$ is the dummy variable:

$$I_{t-1}=\left\{\begin{array}{c} 1\;when\;\mu_{t-1}<0\;shows\;postive\;shocks\\ 0\;when\;\mu_{t-1}\geq 0\;shows\;negative\;shocks \end{array}\right\}$$

Where the symbol $\sigma_{t}^{2}$ refers to the conditional variance, ω is the constant term, $u_{t-1}^{2}$ and $\sigma_{t-1}^{2}$ represent the return square at time t−1, and conditional variance at time t−1. Whereas, γ refers to the leverage effect coefficient.

## 4 Empirical analysis

Table 2 refers to the descriptive statistics of daily stock indices returns of the developed markets, it can be noted that before the COVID-19 pandemic the mean values for all the stock markets are positive except TA 35 whereas, during the COVID-19 pandemic, the mean return values for most of the stock markets are negative, which means that COVID-19 crisis has a bearish trend in most of the developed stock markets. Furthermore, the values for the measure standard measure of volatility (i.e., standard deviation) of all the stock returns during the COVID-19 are greater than before the COVID-19 period. Thus, it can be concluded that stock markets volatility has increased during the COVID-19. Additionally, the higher moment's skewness and kurtosis values show that for both the periods the stock returns are asymmetrically distributed, these outcomes are further confirmed via Jarque-Bera test that stock returns do not follow the patterns of normal distribution.

**Table 2:** Descriptive statistics for developed stock indices.

Before COVID-19 period						During COVID-19 period				
Stock Index	Mean	Standard Deviation	Kurtosis	Skewness	Jarque-Bera	Mean	Standard Deviation	Kurtosis	Skewness	Jarque-Bera
ASX 200	0.000735	0.010253	3.34939	−2.51537	1649.68	−0.00057	0.020439	4.71624	−0.76248	173.65
ATX	0.000667	0.011592	5.36487	−2.94536	2923.21	−0.00127	0.025452	7.68190	−0.71144	432.29
BEL 20	0.001015	0.011882	7.76335	−2.39376	991.26	−0.00062	0.022764	9.03484	−1.34542	631.67
S&P/TSX	0.000929	0.010015	8.9038	−6.04321	27515.52	−0.00048	0.02265	12.2778	−0.51833	1077.39
OMXC20	0.001514	0.011129	2.64001	−0.72235	63.93	−0.000326	0.033569	5.22748	−0.81015	211.9
OMX Helsinki 25	0.001516	0.011245	3.49354	−1.98098	754.8	−0.00062	0.038794	5.74687	−0.76483	250.19
CAC 40	0.0012	0.011943	5.74587	−2.71045	1559.25	−0.00094	0.022072	6.60776	−0.69666	322.69
DAX	0.00153	0.011812	2.40793	−2.34652	1086.25	−0.00091	0.032409	7.55900	−0.43169	409.5
FTSE China 50	0.000998	0.011446	1.85607	−0.74926	40.34	−0.0003	0.026838	2.20988	0.13979	34.36
ISEQ All Share	0.001386	0.012176	3.67237	−1.06429	187.09	−0.0003	0.021164	4.69968	−0.87087	177.8
TA 35	−0.0002	0.008548	2.03374	−1.78252	443.81	−0.00016	0.029237	17.5252	−2.15806	2320.93
Nikkei 225	0.00157	0.010038	3.50820	−1.14886	181.97	−0.00044	0.036902	4.88442	0.54656	176.71
AEX	0.001365	0.011344	6.22732	−2.54922	1253.46	−0.00081	0.028777	7.99643	−0.61739	463.61
NZX 50	0.000929	0.007685	1.46305	−0.56439	24.02	−0.000253	0.024594	8.04794	−0.46626	464.62
OSE Benchmark	0.001308	0.011927	3.19414	−2.44781	1413.29	−0.00088	0.037231	4.50095	−0.85960	164.28
PSI 20	0.000375	0.011215	6.3777	−3.11499	2957.25	−0.00105	0.026993	8.062046	−0.47458	466.47
IBEX 35	0.00064	0.011737	7.52283	−2.40365	1113.05	−0.00113	0.023022	9.017942	−0.91586	600.68
OMXS30	0.001436	0.005479	1.1513	11.89814	173645.02	−0.000119	0.029294	5.676669	−0.85075	248.62
SMI	0.000891	0.009768	3.93873	−2.02748	567.72	−0.0004	0.025735	10.04676	−0.8645	737.4
FTSE 100	0.000575	0.010927	4.349	−2.55765	1458.85	−0.00121	0.029671	6.8536	−0.58629	341.98
Dow 30	0.001212	0.012776	4.9107	−1.07898	878.52	−0.00028	0.024939	8.34097	−0.4635	498.66

**Note:** ASX = Australia, ATX = Austria, BEL 20 = Belgium, S&P/TSX = Canada, OMXC20 = Denmark, OMX Helsinki 25 = Finland, DAX = Germany, FTSE China 50 = Hong Kong, ISEQ All Share = Ireland, TA 35 = Israel, Nikkei 225 = Japan, AEX = Netherlands, NZX 50 = New Zealand, OSE Benchmark = Norway, PSI 20 = Portugal, IBEX 35 = Spain, OMXS30 = Sweden, SMI = Switzerland, FTSE 100 = United Kingdom, Dow 30 = United States, CAC 40 = France.

Table 3 shows the results of the Augmented Dickey-Fuller test and the ARCH-LM test. All the stock indices return in level form has a greater test value than a critical threshold, resulting in the rejection of the null hypothesis i.e., the presence of unit root is rejected, hence, it can be concluded that stock market indices are stationary in their level form at 1 %. Furthermore, the p-values of LM statistics suggest the rejection of the null hypothesis i.e., there is no ARCH effect, thus there exists ARCH effect in the residuals of the stock indices returns. Hence, all the pre-requisites for using GJR-GARCH [1,1] model are fulfilled.

**Table 3:** Augmented Dickey-Fuller test and the ARCH-LM test.

Stock Markets	ADF in Level T-statistics	ARCH Effects Obs *R-squared
ASX 200	−19***	39.66***
ATX	−10.9**	53.05*
BEL 20	−5.8***	8.89***
S&P/TSX	−6.9*	68.35***
OMXC20	−14.5***	1.96***
OMX Helsinki 25	−8.8**	8.37***
CAC 40	−8.8***	9.26**
DAX	−8.7***	13.72***
FTSE China 50	−7.9*	54.27***
ISEQ All Share	−7.6***	50.42***
TA 35	−8.4***	12.11**
Nikkei 225	−8.9***	59.57***
AEX	−9.5***	11.9***
NZX 50	−9***	44.65***
OSE Benchmark	−15.7***	25.93***
PSI 20	−7**	17.45*
IBEX 35	−6.9***	10.4***
OMXS30	−7.8***	11.07***
SMI	−4.8***	12.51**
FTSE 100	−6.4***	10.19***
Dow 30	−6.8**	78.95***

Note: *** refers to 1 % significance level, ** shows the 5 % significance level, * refers to 10 % significance level.

Table 4, illustrates the empirical results of GJR-GARCH for all the selected stock indices before the COVID-19 period. The conditional mean parameter for all the indices is significantly positive. Moreover, in the variance equation, all the coefficients including constant variance term, ARCH, and GARCH parameters are significantly positive. The parameters of ARCH effect (α), and GARCH effect (β), refer to the news related to the financial markets. Particularly, α denotes the recent news whereas, β shows the old news, and both the parameters are statistically significant which means both the new and old news affects the stock market volatility. Moreover, higher the value of GARCH coefficient means that it takes longer period of time for shock in returns series to die away, in other words it signifies that volatility in the stock returns is persistent. Additionally, Table 4 shows that the sum of both the parameters (α+β) for the selected stock indices returns ranges from 0.781 to 0.984, and most of the stocks returns are close to the value of one which means returns volatility for the selected indices shows persistent behavior. Moreover, the sum of (α+β) for all the stock indices is less than 1, which indicates that the stock returns follow the process of mean reversion i.e., returns tend to revert to their mean values. Hence, among all the developed stock markets, the ASX 200, DAX, and PSI 20 follow the slowest mean reversion process whereas, FTSE China 50, and OMX Helsinki 25 follow the fastest mean-reversion process. In another context similar results were reported by Ref. [33].

**Table 4:** GJR-GARCH (1, 1) results before the COVID-19 period.

Parameters	μ	$\alpha_{0}$	α	β	α+β	γ	Log likelihood	AIC
ASX 200	0.001575	0.000046	0.230***	0.551***	0.781	−0.061479**	480.62	−955.24
ATX	−0.000093	0	0.127***	0.821**	0.948	−0.114815***	408.08	−810.16
BEL 20	0.001054	0.000007	0.180*	0.726***	0.906	0.37726***	427.82	−849.64
S&P/TSX	0.000738	0	0.145***	0.771***	0.916	−0.021082***	482.37	−958.74
OMXC20	−0.000072	0.000037*	0.141**	0.721**	0.862	0.130378***	524.35	−1042.70
OMX Helsinki 25	−0.00005	0.000108	0.143**	0.841***	0.984	0.234051*	464.53	−923.05
CAC 40	0.001534	0.000036	0.114***	0.847*	0.961	0.258452***	433.67	−861.33
DAX	0.00003	0	0.127***	0.721***	0.848	0.116547***	430.60	−855.20
FTSE China 50	0.000627	0.000008	0.131***	0.847***	0.978	0.095304***	489.25	−970.50
ISEQ All Share	0.000776	0	0.110*	0.847***	0.957	0.412796***	441.84	−877.67
TA 35	−0.000989	0.000033***	0.167**	0.747***	0.914	0.030163**	499.03	−992.05
Nikkei 225	−0.000144	0.000008***	0.170***	0.741**	0.911	−0.072579***	508.12	−1010.23
AEX	0.001244	−0.067	0.115***	0.747*	0.862	−0.152924***	462.70	−917.41
NZX 50	0.000001	−0.727561***	0.125*	0.847**	0.972	0.47836***	528.16	−1050.31
OSE Benchmark	0.000445	−0.423212**	0.132***	0.761***	0.893	−0.032132***	491.48	−976.96
PSI 20	0.000563	−0.300879***	0.116**	0.741**	0.857	0.143347***	481.23	−956.45
IBEX 35	−0.001059	−0.220348***	0.147**	0.755***	0.902	0.543251**	428.20	−850.41
OMXS30	0.001575	−1.745254	0.146***	0.822*	0.968	0.449452**	460.05	−914.10
SMI	−0.000093	0.000046	0.162***	0.771*	0.933	0.226547***	499.69	−993.38
FTSE 100	0.001054	0	0.153*	0.747***	0.900	0.125304***	457.17	−908.35
Dow 30	0.000738	0.000007	0.116*	0.846**	0.962	0.641796***	449.38	−892.75

**Note:** *** refers to 1 % significance level, ** shows the 5 % significance level, * refers to 10 % significance level.

Table 5, shows the GJR-GARCH [1,1] results during the COVID-19 period. It can be seen that the conditional mean parameter for all the stock indices is negative which indicates that during the COVID-19 period, the developed stock markets have followed a bearish trend. As compared to the before COVID-19 period, the ARCH **(**α), and GARCH (β) parameters along with their sum **(**α+β**)** for the stock returns are higher during the COVID-19 period. Thus, the stock indices show more persistence in their volatility during the COVID-19 period, and the stock returns also follow the faster mean reversion process as compared to the before the COVID-19 period. Moreover, the same results were reported by Ref. [14]. Furthermore, the gamma coefficient for all the stock indices is positive except ASX 200 and AEX, which indicates during the COVID-19, all the stock markets exhibit a significant asymmetric leverage effect in their returns. Overall, it can be concluded that during the COVID-19 period, stock returns have shown increased persistence volatility, mean reversion phenomena, and leverage effects.

**Table 5:** GJR-GARCH (1, 1) results during the COVID-19 period.

Parameters	μ	$\alpha_{0}$	α	β	α+β	γ	Log likelihood	AIC
ASX 200	−0.00057	0.00021**	0.130***	0.861***	0.991	−0.033637	485.62	−953.24
ATX	−0.00127	0.00064*	0.126***	0.811***	0.937	0.012225	4010.08	−808.16
BEL 20	−0.00062	0.00052***	0.166**	0.781***	0.947	0.19395**	429.82	−847.64
S&P/TSX	−0.00048	0.00025	0.244***	0.741**	0.985	0.011201	485.37	−956.74
OMXC20	−0.000326	0.00016	0.141***	0.817***	0.958	0.296711***	529.35	−1040.70
OMX Helsinki 25	−0.00062	0.00032**	0.112**	0.876*	0.988	0.018919	468.53	−921.05
CAC 40	−0.00094	0.00047*	0.161***	0.821***	0.982	0.191335***	438.67	−859.33
DAX	−0.00091	0.00052***	0.127**	0.791**	0.918	0.136151***	435.60	−853.20
FTSE China 50	−0.0003	0.00020	0.131***	0.831*	0.962	0.367853***	494.25	−970.50
ISEQ All Share	−0.0003	0.00043**	0.147***	0.793***	0.94	0.138397	449.84	−875.67
TA 35	−0.00016	0.00020*	0.103***	0.847***	0.95	0.250228***	509.03	−990.05
Nikkei 225	−0.00044	0.00012***	0.127***	0.822***	0.949	0.207247*	514.12	−1008.23
AEX	−0.00081	0.00035	0.116***	0.861***	0.977	−0.444637	467.70	−919.41
NZX 50	−0.000253	0.00011	0.113***	0.831***	0.944	0.222435	536.16	−1048.31
OSE Benchmark	−0.00088	0.00021	0.132**	0.726***	0.858	0.39495**	498.48	−974.96
PSI 20	−0.00105	0.00027**	0.161***	0.829*	0.99	0.411201	488.23	−954.45
IBEX 35	−0.00113	0.00048***	0.117***	0.835***	0.952	0.585711***	438.20	−848.41
OMXS30	−0.000119	0.00033*	0.119***	0.865**	0.984	0.715919	467.05	−912.10
SMI	−0.0004	0.00021	0.114***	0.881***	0.995	0.491535***	504.69	−991.38
FTSE 100	−0.00121	0.00034**	0.187***	0.771***	0.958	0.536151***	459.17	−906.35
Dow 30	−0.00028	0.00033*	0.212**	0.726***	0.938	0.867853***	456.38	−890.75

**Note:** *** refers to 1 % significance level, ** shows the 5 % significance level, * refers to 10 % significance level.

## 5 Conclusion & discussion

The current ongoing COVID-19 pandemic has affected the world economy including, financial markets such as stock markets. Subsequently, stock markets exhibit a high level of volatility during this pandemic. Therefore, the current study models and analyses the influence of COVID-19 on the stock markets of developed markets. The daily stock indices return data was considered over the period of 01-July-2019 to 18-November-2020. The descriptive statistics showed that during the COVID-19 period, all the stock indices gave negative returns whereas, before the COVID-19 period the stock indices gave positive returns. Furthermore, the empirical findings from the GJR-GARCH [1,1] model reveals that all the selected stock indices showed leptokurtic phenomena, volatility clustering, and leverage effect in both the before period, and during the COVID-19 period. Additionally, the conditional volatility of all the stock indices is influenced by both the recent news (ARCH effect), and old news (GARCH effect). However, these financial stocks tend to vanish over time, hence the developed stock markets volatility exhibit “persistent behavior.” These results are inline with the findings of [37].

Overall, this study investigated the volatility of developed stock markets before and during the COVID-19 pandemic using daily stock indices return data from 21 developed markets over the period of 01-July-2019 to 18-November-2020. The study found that all the stock indices gave negative returns during the COVID-19 period, while they were positive before the pandemic. The empirical findings from the GJR-GARCH [1,1] model revealed that all the selected stock indices showed leptokurtic phenomena, volatility clustering, and leverage effect in both the before and during the COVID-19 period. The study also showed that the persistence behavior of the developed stock markets' volatility tends to vanish over time. Similar results were reported by Ref. [38]. Moreover, the study emphasized the impact of the COVID-19 pandemic on global stock markets and warned policymakers to be cautious since the variants of COVID-19 change with almost every wave and may lead to another financial crisis. Therefore, economic policymakers must focus on economic indicators that are significant in decreasing the volatility in the stock markets.

Moreover, the empirical findings of the study have proved the impact of the COVID-19 pandemic on global stock markets. Besides, policymakers need to be cautious because the variants of COVID-19 change with almost every wave. Hence, it can lead the global economy to another financial crisis. Thus, in order to minimize the financial risk associated with the crises, economic policymakers must focus on economic indicators that are significant in decreasing the volatility in the stock markets. Accordingly, the outcomes of our study would help government policymakers to investigate the economic and financial factors that are of use to counter the current pandemic.

### 5.1 Financial implications

The results extracted also provide useful financial implications for potential investors which can benefit them in making effective decisions related to portfolio management. In general, COVID-19 pandemic has strongly affected the returns in the financial markets whereas, stock market returns in particular, thus making them more volatile. For that purpose, investment managers should focus on the potential effects of COVID-19 while constructing portfolios for new investments. The analysis of developed stock markets during the crisis is used for the future volatile behavior of their returns. The results suggest that COVID-19 has made the stock markets of developed economies more volatile. For that purpose, investors particularly in the developed economies can utilize the information regarding the financial impacts of COVID-19 pandemic.

Furthermore, the identification of increased volatility persistence and the presence of leptokurtic phenomena during the COVID-19 pandemic period underscore the need for robust risk management strategies. These insights can guide investors in implementing effective risk mitigation techniques and constructing resilient portfolios that incorporate assets with low correlation to stock markets. Moreover, the awareness of the leverage effect allows for better assessment and management of exposure to potential downside risks. The study also highlights the potential investment opportunities that arise during periods of market volatility and emphasizes the importance of maintaining a long-term perspective. By considering these implications, investors can navigate turbulent market conditions, effectively manage risks, and capitalize on suitable investment prospects.

### 5.2 Limitations

Considering the limitations, this study sets up directions for future researches. First, this empirical research is restricted to the stock markets of developed economies, in future other economies such as emerging, and frontier stock markets can be included. Second, the domain of this study covers the stock markets in particular, upcoming studies should increase their scope by examining the volatility of other financial markets, such as foreign exchange, cryptomarket, and bond market etc. Third, more rigorous techniques and asymmetric volatility models can be applied in a form of threshold ARCH (TARCH), which consists of signs indicating the volatilities invention, which can influence the stock returns fluctuations.

## Author contributions

Muhammad Irfan: Writing – review & editing, Visualization, Validation, Supervision, Software, Resources, Project administration, Methodology, Investigation, Funding acquisition, Formal analysis, Data curation, Conceptualization. Ahmet Faruk Aysan: Writing – review & editing, Writing – original draft, Visualization, Validation, Supervision, Software, Resources, Project administration, Methodology, Investigation, Funding acquisition, Formal analysis, Data curation, Conceptualization. Mrestyal Khan: Writing – review & editing, Writing – original draft, Visualization, Validation, Supervision, Software, Resources, Project administration, Methodology, Investigation, Funding acquisition, Formal analysis, Data curation, Conceptualization. Maaz Khan: Writing – review & editing, Writing – original draft, Visualization, Validation, Supervision, Software, Resources, Project administration, Methodology, Investigation, Funding acquisition, Formal analysis, Data curation, Conceptualization. Roohi Mumtaz: Writing – review & editing, Visualization, Validation, Supervision, Software, Resources, Project administration, Methodology, Investigation, Funding acquisition, Formal analysis, Data curation, Conceptualization. Umar Nawaz Kayani: Writing – review & editing, Writing – original draft, Visualization, Validation, Supervision, Software, Resources, Project administration, Methodology, Investigation, Funding acquisition, Formal analysis, Data curation, Conceptualization

## Data availability statement

Data can be downloaded from www.investing.com.

## Declaration of competing interest

The authors declare that they have no known competing financial interests or personal relationships that could have appeared to influence the work reported in this paper.
